# Impact of Healthcare on Stock Market Volatility and Its Predictive Solution Using Improved Neural Network

**DOI:** 10.1155/2022/7097044

**Published:** 2022-08-11

**Authors:** Nusrat Rouf, Majid Bashir Malik, Sparsh Sharma, In-Ho Ra, Saurabh Singh, Abhishek Meena

**Affiliations:** ^1^Department of Computer Sciences, Baba Ghulam Shah Badshah University, Rajouri 185234, India; ^2^Department of Computer Science & Engineering, National Institute of Technology, Srinagar, Jammu and Kashmir, India; ^3^School of Computer Information and Communication Engineering, Kunsan National University, Gunsan, Republic of Korea; ^4^Department of Industrial & Systems Engineering, Dongguk University, Seoul 04620, Republic of Korea; ^5^Division of Physics and Semiconductor Science, Dongguk University, Seoul 04620, Republic of Korea

## Abstract

The unprecedented Corona Virus Disease (COVID-19) pandemic has put the world in peril and shifted global landscape in unanticipated ways. The SARSCoV2 virus, which caused the COVID-19 outbreak, first appeared in Wuhan, Hubei Province, China, in December 2019 and quickly spread around the world. This pandemic is not only a global health crisis, but it has caused the major global economic depression. As soon as the virus spread, stock market prices plummeted and volatility increased. Predicting the market during this outbreak has been of substantial importance and is the primary motivation to carry out this work. Given the nonlinearity and dynamic nature of stock data, the prediction of stock market is a challenging task. The machine learning models have proven to be a good choice for the development of effective and efficient prediction systems. In recent years, the application of hyperparameter optimization techniques for the development of highly accurate models has increased significantly. In this study, a customized neural network model is proposed and the power of hyperparameter optimization in modelling stock index prices is explored. A novel dataset is generated using nine standard technical indicators and COVID-19 data. In addition, the primary focus is on the importance of selection of optimal features and their preprocessing. The utilization of multiple feature ranking techniques combined with extensive hyperparameter optimization procedures is comprehensive for the prediction of stock index prices. Moreover, the model is evaluated by comparing it with other models, and results indicate that the proposed model outperforms other models. Given the detailed design methodology, preprocessing, exploratory feature analysis, and hyperparameter optimization procedures, this work gives a significant contribution to stock analysis research community during this pandemic.

## 1. Introduction

The stock market plays an important role in country's economy, as it is a platform for most of the money exchange throughout the world. Investing in a stock market through a disciplined strategy can lead to substantial gains. The stock market prediction is a challenging task due to the nonlinear, dynamic, and chaotic data [1]. A wise investment is only possible if the stock data is analyzed properly before investment. The analysis of the voluminous volatile and dynamic financial data is challenging. With the advent of online trading, people are moving towards the automated intelligent decision support systems rather than using classical fundamental analysis approaches for stock price prediction [2, 3].

The global economy and stock markets have been greatly affected by COVID-19. Significant market indexes, such as the Standard and Poor's (S&P 500), plummeted by 41% on February 19, 2020 [4]. Economic operations of numerous countries suddenly halted as tight quarantine rules were implemented to combat the devastations of this outbreak [5]. Transportation between countries has been limited, leading to the hampering of global economic activity [6]. Global markets have labelled this disaster a black swan event. This pandemic has significantly disturbed and influenced stock market activity around the world [7]. In year 2020, due to the effects of pandemic, it was estimated that the China's Gross Domestic Product (GDP) may fall by 6.2 percent, while the United States (US) GDP may plunge down by 8.4 percent [2, 8]. The rest of the world's currency could lose 5.9% of its value. Due to the increased volatility, foreign investors have been withdrawing money from the markets that has weakened the economy [9, 10]. Panic selling has induced confusion among investors. Therefore, predicting the markets in such conditions is challenging. The stock index is a representative of whole stock market or a sector or a part of market. It is a temporal characteristic with low signal-to-noise ratio and heavy-tailed distribution [11]; thus, prediction is a challenging task. Prediction of index prices aims to build a model where the independent variable is predicted using numerous dependent variables. Artificial Neural Network (ANN) models are the most popular method that adapt to the dynamic nature of markets easily [12]. These are the models that understand the context of a problem by creating multiple linear transformations on the feature space followed by a nonlinearity to create its simplified representation [7, 13]. Neural network models can represent any distribution over the input feature space; therefore, they are known as universal function approximator [14]. Neural networks prove to be a better option when there is a nonlinear relationship between independent and the dependent variables [15]; thus, they can predict stock index prices to a good extent.

In this study, a novel approach for stock price prediction is implemented in which a customized neural network is developed and the power of hyperparameter optimization in modelling stock index prices is investigated. A novel dataset is generated using nine standard technical indicators and COVID-19 data. In addition, the importance of selecting significant features is emphasised. A novel feature selection approach is developed in which multiple feature ranking algorithms are used for optimal feature selection. The extensive hyperparameter optimization procedures are employed for optimal predictions. Moreover, the model is evaluated by comparing it with other models, and results show that the proposed model outperforms other models.

Some of the notable contributions of this study are as follows:

- A novel dataset has been generated using technical indicators and COVID-19 data.
- An exhaustive hybrid feature engineering has been performed where five feature selection techniques have been applied to the data.
- An improved ANN has been built and trained using an automatic hyperparameter tuning procedure.
- The performance of the proposed model has been compared to other models.

## 2. Background

**Figure 1:**
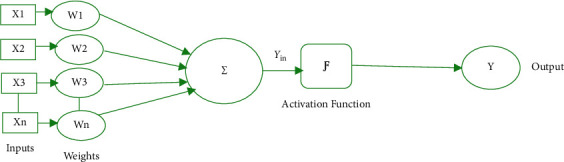
Structure of a neuron.

The ANN is one of the popular and intelligent techniques that has a capability to adapt to the dynamic nature of nonlinear data [16]. It is one of the bioinspired algorithms that mimics the function of brain. By detecting the patterns in data, ANN gathers knowledge and learns through experience [17, 18]. ANN is typically a layered network consisting of one or more artificial neurons or processing elements. The interconnection of the neurons contributes to the computational power of ANN. Each neuron comprises of weighted inputs, an activation function, and an output.  Figure 1 presents the typical structure of neuron. The weighted sum of inputs is passed through activation function to generate the output. The activation function leads to nonlinearity in network [19]. As shown below, *Y*_in_ is the weighted sum of inputs and the output *Y* is the function of *Y*_in_.

(1) $$\begin{array}{c} Y_{\mathrm{in}}=X_{1}.W_{1}+X_{2}.W_{2}+X_{3}.W_{3}+\ldots X_{n}.W_{\text{n}}\text{.}\\ \text{That is net input }Y_{\mathrm{in}}=\sum_{i}^{n}X_{i}.W_{i},\\ Y=F\left(Y_{\mathrm{in}}\right). \end{array}$$

### 2.1. Feed Forward Neural Networks

**Figure 2:**
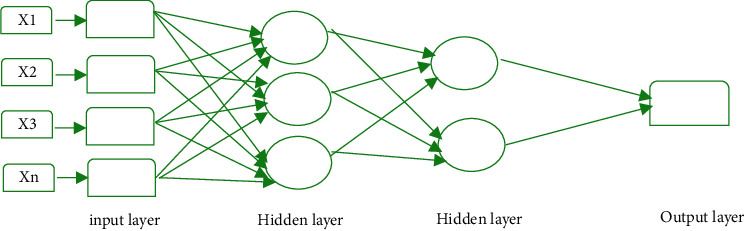
Structure of a feed forward neural network.

Feed Forward Neural Networks (FFNNs) are the simplest type of ANN where the processing is done in only one direction (forward) with no cycles or loops [20, 21]. The processing of input data starts with input layer and passes through hidden layers and then to output layer. The construction of FFNN can be done from different units like binary McCulloch–Pitts neurons [22, 23]. An example of an FFNN is a perceptron.  Figure 2 presents the structure of simple FFNN.

### 2.2. The Error Back Propagation

**Figure 3:**
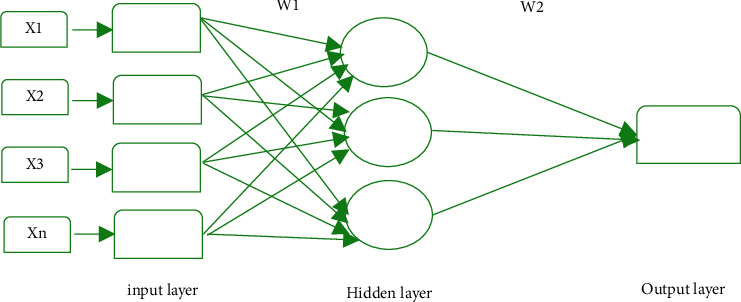
The error back propagation in neural networks.

It is a multilayer FFNN with a series of hidden interconnected layers containing an arbitrary number of neurons. Back Propagation (BP) represents how the error that comes at the last layer of neural network is percolated back so that each of the individual layers can be trained [24]. In the training process, weights are adjusted in a way to reduce the error in predictions to a minimal value. Through BP, errors are minimized by updating the weights and minimizing the cost function using the gradient descent approach [25, 26].  Figure 3 presents the BP neural network having three layers: input, one hidden, and output layer. The nodes are connected through the links.

Between the input and hidden layer weights are *W*1 and between the hidden layer and output layer weights are *W*2. The last layer parameters can be trained directly as they directly impact the loss function but to train the weights of previous layers the feedback needs to travel backwards [27]. The sequence in which weights will be updated is from *W*2 to *W*1. Weights of *W*2 will be updated as follows:

(2) $$\begin{array}{c} W2=W2-\alpha *\Delta W2, \end{array}$$

where Δ*W*2 is (*Y*′ − *Y*)^2^ and for *W*1 the weights will be updated as follows:

(3) $$\begin{array}{c} W1=W1-\alpha *W1, \end{array}$$

where Δ*W*1 is directly proportional to Δ*W*2, which means that any change in Δ*W*2 will introduce a change in Δ*W*1.

### 2.3. The Loss Function

At the heart of any machine learning algorithm is a loss function, which is the sum measure of errors that we are trying to minimize. A way to minimize this loss is to find out the point over the function where it exhibits the minimum value and gradient/error tends to zero. The slow clipping away of the path to minima is known as gradient descent. In regression problem, the mean squared error is usually chosen as a loss function [28]. The generalized formula is given in the equation below.

(4) $$\begin{array}{c} J=\left(\frac{1}{2n}\right)*\sum_{j=1}^{n}{\left[Y_{j}^{\prime }-Y_{j}\right]}^{2}. \end{array}$$

*J* is the loss function wherein for each example starting from *j* = 1 to *n* from true value the predicted value is subtracted and squared and added across all the examples, and it is divided by 2*n*, where *n* is the number of examples, where *Y*_*j*_′ is true value and *Y*_*j*_ is predicted value.

(5) $$\begin{array}{c} Y_{j}=F{\left(\sum_{i=1}^{m}X_{i}\cdot W_{i}\right)}^{2}. \end{array}$$

*Y*_*j*_ or predicted value is given by summation from 1 to *m*, where *m* is the number of features and multiplying each feature with corresponding weights and adding it.

If we have to find the optimum parameters which are typically the weights that are between neurons. The loss function will be decreased by decreasing these weights by a factor of alpha (*α*) multiplied by (*∂J*/*∂W*) as shown in the following equation.

(6) $$\begin{array}{c} W=W-\alpha *\frac{\partial J}{\partial W}, \end{array}$$

where *α* is the learning rate and *J* is the cost function. At the end, the objective is to minimize the loss function constrained on *W* given as

(7) $$\begin{array}{c} \text{argmin }J⟶\text{optimized weights }W. \end{array}$$

### 2.4. The Weight Initialization

There are various methods for initializing weights in neural networks [29]. To begin with, zero initialization initializes all weights to zero. It does not give good results because all weights start at the same point. So, it takes longer time to converge to minima because of lack of generalization [30]. In normal initialization, weights are sampled from a normal distribution. It gives better results and tends to provide more room for exploration in terms of feature space. Glorot/Xavier initialization is variation of normal initialization where the weights are initialized from a normal distribution centered at 0 and variance is 2/(fan_in +fan_out) [31]. Fan-in is the number of nodes in previous layer and fan-out is the number of nodes in next layer. This initialization gives good results and helps to converge quickly by taking into consideration the architecture of network. In the similar line is He initialization, which is a random initialization depending on the size of previous layer and helps to attain global minimum of cost function faster and more efficiently [30].

## 3. Related Work

A wide range of problems are solved using ANNs due to its versatile nature. With the introduction of multilayer concept, ANNs are getting more attention for prediction besides other methods. ANNs are built primarily on the features extracted from input data. ANNs deal with the nonlinear relationships of time series data by learning from experience and building complex neural networks to generate optimal solutions for prediction problems [32, 33]. ANN consists of input layer, zero or more hidden layers, and an output layer. It is one of the frequently used machine learning algorithms that is applied to time series data for stock trend, index, and market predictions [34]. Some of the studies on stock market predictions using ANNs are discussed below.

Yudong and Lenan [35] developed a BP ANN model where weights are updated using IBCO tool to predict the next day and 15-day ahead price movement of stock index. The stock data of Standard and Poor's (S&P500) of the United States of America (USA) is used along with the technical indicators. The model is compared with {9-3-1}, and the proposed method achieved significant results.

Bijari and Bijari [36] developed a hybrid ANN where the model follows two phases. In the first phase, an Autoregressive Integrated Moving Average (ARIMA) model is used to extract essential features from time series data. In the second phase, ANN model is used to generate predictions from extracted data. The model is applied to three well-known datasets: the Canadian lynx data, the US dollar exchange rate data, and the Wolf's sunspot data. The model performance is calculated using MSE and Mean Absolute Error (MAE). Comparison is done with the ANN, ARIMA, and Zhang's model [37]. The proposed model achieved a better accuracy as compared to other mentioned models.

Kara et al. [38] compared the performance of two models, ANN and SVM, in predicting the daily direction of ISE National 100 index. The authors used ten technical indicators and extensive parameter tuning for models was performed to improve prediction accuracies. The average performance of SVM was around 71% and for ANN was around 75%, which is significantly better than the former.

Guresen et al. [39] compared the three stock market prediction models, namely, multilayer perceptron (MLP), dynamic artificial neural network (DAN2), and hybrid neural networks, which use generalized autoregressive conditional heteroscedasticity (GARCH) to generate new input variables [39]. Each model was evaluated on two points: MSE and Mean Absolute Deviate (MAD). The models were applied to NASDAQ stock data. The results showed that MLP outperformed DAN2 and GARCH-MLP.

Bing et al. [40] did an extensive comparison between eight models for prediction of stock value of central bank of Turkey. The six models were based on ANNs, where the number of hidden layers was changed in each model and other two models were based on moving averages. The models were evaluated using coefficient of determination. The ANN models with one hidden layer outperformed all models.

Yetis et al. [21] used generalized feed forward neural networks to predict the stock value of NASDAQ index. The model was trained on the one-year share market data of NASDAQ [21]. The model performed best with five parameters and 10 neurons in input layer. The performance of model was calculated using MSE. Furthermore, the authors have drawn a conclusion that when stock index prices are greater than 3000, the error reduces to less than 2%.

Patel et al. [41] compared the two approaches to predict the stock values of two market indices (CNX Nifty and S&P Bombay Stock Exchange Sensex) and two companies (Reliance Industries and Infosys Ltd.). The market data from 2003 to 2012 is extracted. The first approach focuses on the calculation of ten technical indicators from market data while in the second approach, the technical indicators are represented as trend determination layer. Four models, ANN, SVM, random forest, and naïve Bayes, are applied. In this first approach, random forest outperforms all other models and in the second approach the performance of each model increases.

Wang et al. [42] proposed a hybrid model that combines the powers of Elman recurrent neural network with stochastic time effective function. The model was tested on four indices: SSE, TWSE, KOSPI, and Nikkei225. The performance of model was compared with stochastic time effective neural network, backpropagation neural network, and Elman recurrent neural network. From the empirical results, it was revealed that the proposed model outperformed all other mentioned models.

Hajirahimi and Hajirahimi [43] compared the predictive power of five hybrid models using serial and parallel approaches. Two serial hybrid models ARIMA-MLP and MLP-ARIMA and three parallel models based on simple average, genetic algorithm, and linear regression were applied to two datasets SZII and S&P 500. The results indicate that all hybrid models with serial combinations outperformed other hybrid models.

Chopra et al. [34] investigated the prediction ability of ANN before and after demonetization in India. The ANN with Levenberg–Marquardt algorithm is applied to the data of nine companies and stock index CNX Nifty50. The model performance is measured using MSE. The regression value of 0.99 is achieved, which depicts the efficiency of model.

Vijh et al. [15] compared the ANN and Random Forest models to predict the next day stock prices of five companies. The market data is used to generate the technical indicators RMSE and MBE and MAPE metric is used to evaluate the models. ANN outperforms RF and obtains low values of performance metrics.

The following are the few papers that have analyzed the impact of COVID-19 on stock markets:

Zeren and Hizarchi [44] have used regression method to investigate the effect of COVID-19 on the stock market and discovered a significant relationship between COVID-19 variables and stock returns. Aravind and Manojkrishnan [45] investigated the association between pharmaceutical stocks and COVID-19 and found that pharmaceutical stocks had a negative reaction. Zhang et al. [46] investigated the effect of COVID-19 on financial markets using statistical analysis revealed that COVID-19 has raised global financial market risk. Individual stock market volatility is directly proportional to the intensity of viral outbreak in that country. Goodell [47] analyzed a large body of material on the economic impact of natural disasters and noted that the global economic destruction caused by COVID-19 is unparalleled. Baker et al. [48] used text analysis and other methods to investigate the influence of COVID-19 news on stock market volatility. The results revealed that COVID-19 affected the markets to greater extent than similar other diseases. Bekhet et al. [5] have developed a technique for early COVID-19 detection using medical experiences. The hyperparameter optimization of light-weight Convolution Neural Networks has been done and authors achieved an accuracy rate of 96% on some benchmark datasets. Ibrahim et al. [6] have developed a hierarchical twitter sentiment model (HSTM) to analyze the sentiment of people on High Tech Computer (HTC) product and COVID-19 in Twitter messages without predetermining the depth and width hierarchy of first discovered hierarchical tree. The authors have employed valence-aware dictionary and sentiment reasoner (VADER) sentiment analyzer to analyze sentiments of people and have grouped sentiments into groups like positive, negative, strongly positive, and strongly negative. The results show that HSTM analyzes the sentiments of people in an easy and effective way.

Some of the conclusions that can be drawn from the surveyed literature are as follows:

1. It is evident from the surveyed literature, not much work has been done on impact of COVID-19 on stock markets. Mostly, either statistical analysis or regression analysis has been used in the literature so far.
2. Identification of determining features is imperative as it may increase the prediction accuracies [41]. Moreover, all the presented works follow a certain feature selection algorithm and select the features that are best voted. However, each algorithm has a selection criterion, which may or may not benefit your model. Therefore, relying on a single feature selection algorithm is not a good practice.
3. Moreover, technical indicators have a significant impact on investment decisions [49]. Therefore, it is important to consider the preprocessing and importance of standard technical indicators.

Thus, in this study, an idea is devised to derive the important technical indicators from the existing market data along with the COVID-19 data and apply multiple feature selection approaches to generate the important features so that the model prediction accuracy increases significantly. Furthermore, the hyperparameters govern the overall training process, so it is imperative to understand the hyperparameters and fine-tune them to get optimal predictions.

## 4. Methodology

This work attempts to address three research questions:

- What is the effectiveness of COVID-19 and derived technical indicators on the prediction model?
- How effective is feature engineering in increasing the model performance?
- How the hyperparameter optimization procedures effect the model performance?

**Figure 4:**
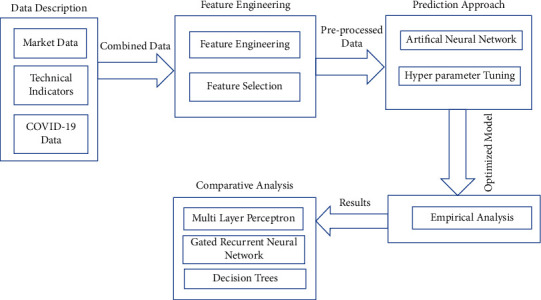
Methodology for stock market prediction.

Our first question is based on the effectiveness of the COVID-19 and technical indicators. As seen from the previous works, technical indicators play a significant role in predicting the markets [50]. Also, COVID-19 has significantly affected the financial markets [7]. In our approach, an attempt has been made to predict the Nifty50 index prices using basic market data COVID-19 data and derived technical indicators. Second research question is based on the effectiveness of feature engineering. From the previous works, it can be concluded that stock data is chaotic and features are highly correlated, which makes it difficult to predict markets with significant accuracies. Therefore, it is essential to apply feature engineering procedures to stock data so that the results may get optimized. In our approach, multiple feature selection models have been applied to select the best features and the average means of coefficients of all models have been used to select the features that have higher average impact on dependent variable. Furthermore, we check how the selection of different hyperparameters impact the model accuracies.  Figure 4 presents the methodology of the work carried out.

### 4.1. The Dataset Description

This section provides the detailed introduction to research data and extracted technical predictors. The openly available Nifty50 open high, low, close and volume (OHLCV) data is retrieved from the following website (https://finance.yahoo.com) for a period of two years. The choice of choosing two-year time span is due to the reason that investors usually perform stock trend analysis using the recent data mostly within 2 years [51]. Massive volumes of COVID-19-related data are constantly being generated as a result of the rapid growth of big data technologies, making it challenging to access correct information resources [52, 53]. The COVID-19 data has been collected from a reliable source https://www.ecdc.europa.eu/en that gives access to the COVID-19 data for more than 200 virus-affected nations and regions. Furthermore, nine technical variables have been added to the market data. There are a wide range of technical indicators. Some indicators perform better under trending markets, and some are effective under nontrending market [54]. From these previous works, it is concluded that with the addition of technical indicators prediction models perform better [35]. Based on the previous works, we selected nine technical indicators, out of which seven have 4 levels [15, 38, 41].  Table 1 presents the selected technical indicators and their formulas. As the COVID-19 dataset contained the data for more than 200 countries, we extracted the data by setting a query for extraction of “Daily_Cases” of “India” in python. Furthermore, we added a feature “Total_Cases,” which is a cumulative sum of “Daily_Cases.” We merged the two datasets on the basis of a common feature “Date.”

### 4.2. Exploratory Feature Analysis

In this section, first, data cleaning is performed by filling the missing values using averaging method. Furthermore, outliers have been detected using simple statistical method called Interquartile Range (IQR) [55]. IQR is the difference between the first (*Q*1) and third quartiles (*Q*3) of data. For each feature in dataset, the data beyond the range of (*Q*1 − 1.5 IQR to *Q*3 + 1.5 IQR) is possible outlier. The symmetric distribution is visualized using box plots for each column.

### 4.3. Hybrid Feature Selection Mechanism

The feature selection leads to the better performance of prediction models. As analyzed from the previous studies, the noisy stock data makes predictions difficult; it is important to understand the features and structure of data so that predictions can be optimized [38].

We list three primary reasons why feature selection is important. Firstly, with the reduction in the number of features, less chances of model overfitting are there. The second reason is to extensively understand the underlying features and their relationships with the dependent variable. Lastly, it reduces the training time of the algorithm [51].

In this work, five feature selection methods have been employed. A python dictionary is maintained to store the coefficient scores or feature ranks obtained from all the methods applied. The mean coefficient value for each feature in the dictionary is calculated and the features with high mean coefficient values are selected for model training. The most important features have the highest coefficients in the model and least important features have coefficient values tending to zero.

**Figure 5:**
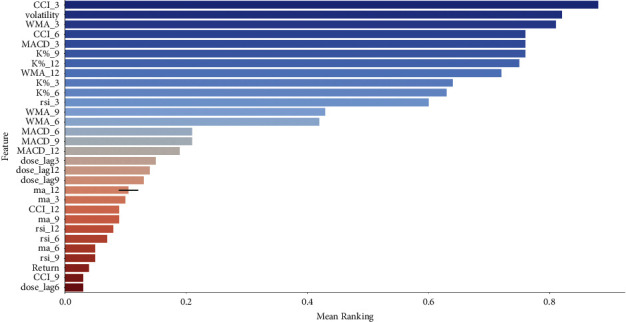
Mean feature rankings.

The models that have been employed are Lasso regression, Ridge regression, Random Forest regression, Stability selection via Randomized Lasso, and Recursive feature elimination (RFE) via Linear Regression. In lasso regression, feature selection is done by picking up the features that have a significant effect on dependent variable. This model is useful for reducing the feature count; however, it fails to interpret the features [56]. Ridge regression model equally distributes the coefficient values between correlated variables [57]. Random forest model with impurity-based ranking selects the top features, and the coefficients of other features aggressively drops off [58, 59]. Stability selection model takes into consideration both the data interpretation and feature importance to improve the models [60]. RFE is widely used algorithm for feature selection. It is based on the greedy optimization to select the significant subset of features [61]. The mean rankings for all the features are visualized in Figure 5. The features are selected where mean ranking is greater than or equal to 0.5. Therefore, out of 30 features, we have selected 10 features as input to our prediction model. Furthermore, it can be analyzed from the Figure 5 that “Daily_cases” feature has a mean feature ranking of greater than 0.5; therefore, it is an important feature and greatly affects the stock market price prediction. Algorithm 1 presents the pseudocode for hybrid feature selection mechanism.

### 4.4. Prediction Approach

ANN is a set of densely interconnected processing units called neurons that are activated through input. The learning mechanism involves the adaption of weights in a way so that prediction error is minimized [62]. In this work, we have employed a four-layer neural network. The input layer has 11 features represented by 11 neurons as input. The output layer has a single neuron with no activation function owing to the fact that it is a regression problem. For each hidden layer, we have employed ReLU activation function. To maintain the balance between variance and bias in a model, it is imperative to select optimal hyperparameters [63].

An extensive hyperparameter tuning has been done for model fitting. The hyperparameters are number of neurons in the input layer, number of hidden layers (*nh*), learning rate (*lr*), number of epochs (*ne*), batch size, dropout, activation function, and shapes of which are discussed as follows: “*nh*” and the number of neurons significantly impact the prediction accuracies. Choosing at least one of the parameters with higher values allows the algorithm to model the relationship between dependent and independent variables effectively. “*lr*” is an essential hyperparameter. If “*lr*” is set high, then the algorithm may adopt to new data quickly but will forget old data and that results in divergence. If “*lr*” is set low, the training algorithm learns slowly and will be less susceptible to outliers and noise [64]. Therefore, we should start with a high “*lr*” value and decrease it till model is optimized. Epoch size represents the number of times the training algorithm will perform through entire training set [65]. Therefore, “*ne*” is an integer that can have values between 0 to infinity. The value of “*ne*” needs to be chosen carefully, as the lower values cause under fitting and higher values cause overfitting due to overoptimizing the weights [63]. Batch size determines learning speed of model and keeps a check on how stable a learning process is. In order to increase the performance of model, the selection of right optimizer is important. So here we have used different variants of stochastic gradient descent (SGD) optimizers like RMSProp, AdaGrad, AdaDelta, and Adam. The description of each variant and differences can be found in [64]. Pseudocode for the feature selection mechanism is presented in Algorithm 1.

Regularization methods enable neural networks to select the models that have efficient generalizing capabilities. Here the idea is to reduce the variance on training datasets, without increasing the bias values. Implementation of regularization using dropout layers is one of the popular techniques. This approach has proven successful, and it can boost the prediction accuracies up to 2% [66]. The value of dropout rate varies between 0 and 1. Whenever there is a possibility of overfitting in model, dropout rate should be increased and conversely, dropout rate should be decreased when model is under fitting the training data [64]. Shapes for hidden layers could be funnel or brick. In funnel shape, the first layer starts with maximum of neurons and subsequently the number of neurons in each layer decreases ending with one neuron in the last layer. While in the brick shape, each hidden layer has the same number of neurons and the last layer contains a single neuron.

To explore the hyperparameter space, different approaches are used like manual search, gird search, and random search. Although, from the empirical evidence, random search method has proven to be efficient in finding the optimal models than grid and manual search approaches [67, 68], however if the dataset is large and complex, this method only explores a tiny hyperparameter space. Moreover, this method is time consuming for high-dimensional data. Therefore, a more efficient solution is to use the python libraries for optimizing the hyperparameters. The main idea is to explore more region of space. The advantage of using these libraries is that it takes care of “zooming” process, which ultimately leads to a better model training in less time [64]. In this work, we have used Talos API for automated hyperparameter optimization that works well on all sorts of complex search spaces [69]. Here the optimal model can be retrieved by running the code with all combinations one time, instead of running code for each combination separately. Furthermore, this library creates an efficient pipeline by following prepare, optimize, and deploy workflow. Therefore, Talos is handy, time-efficient, and powerful method for parameter optimization. Algorithm 2 presents the hyperparameter tuning procedure using Talos.

### 4.5. Performance Analysis

At the heart of any machine learning algorithm is a loss or cost function that calculates the prediction error. The aim is to minimize this error as much as possible. After each iteration, the model calculates the cost function and accordingly adjusts the weights to minimize the error. In our model, we have used mean squared error (MSE), which is the default cost function for regression models, and calculated the average mean of squared differences between actual and predicted values [70]. The MSE ranges between 0 and infinity. The smaller MSE indicates the higher accuracy of models. For a given model, the value of evaluation metrics determines how accurately the model is performing. The model performance is evaluated using percentage-dependent metric, mean absolute percentage error (MAPE) that measures the prediction accuracies, mainly in trend estimation [70]. The MAPE is calculated as follows:

(8) $$\begin{array}{c} \text{MAPE}=\frac{100\%}{n}\sum_{i=1}^{n}|\frac{V_{\text{pred}}-V_{\text{act}}}{V_{\text{act}}}|. \end{array}$$

## 5. Empirical Analysis

**Figure 6:**
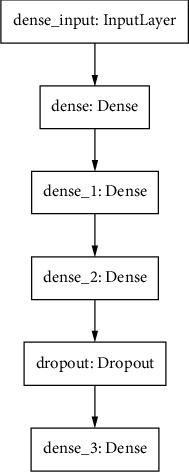
Neural network architecture.

The preprocessed data is divided into training set, test set, and validation set. The 80% of total data is used for training and 50% of remaining 20% is used for test and remaining 50% for validation. The hyperparameters that are used are mentioned in table. The experimentation has been carried out on Google colab with a Python 3 Google Compute Engine backend (TPU). A function has been defined in python to build a basic sequential model using dense and dropout layers. The model is visualized using the pydot library as shown in Figure 6.

### 5.1. Hyperparameter Tuning Analysis

To tune the hyperparameters, we install the Talos library and import the required dependencies. A hyperparameter dictionary is built in which different ranges for hyperparameters are defined for tuning the model. The layout of dictionary is as follows: for “*lr*” ten different values are taken from 0.1 to 0.5 that are equally separated. The number of neurons in input layer is taken as [10, 20], the “*nh*” in the range of 1 to 3, for batch size five different values are taken from 10 to 30, “*ne*” vary from [500, 1000], values of dropout layer are varied between 0 and 0.5, shapes for hidden layer are taken as “brick” or “funnel,” activation function for hidden layers is rectified linear activation function (ReLU) that is nearly linear and has a power of easy optimization with gradient-based methods, and numerous optimizers are applied like RMSProp, AdaGrad, AdaDelta, and Adam.

Table 2 presents the range of values for selected hyperparameters. Instead of passing hypermeters, the dictionary is passed to the model that we built earlier. So here each hyperparameter is defined in a dictionary. The “Scan” function of Talos is used for hyperparameter tuning by passing both the training and validation datasets. This function uses permutation combination of all hyperparameters. The “Analyze” object of Talos carries the results of hypermeter tuning activity. The number of rounds performed for parameter tuning was 2400. To get the lowest validation MAPE value, analyze_object.low() function is used, and it shows that in the 42th round, the lowest validation MAPE of 4.379007 is achieved.

**Figure 7:**
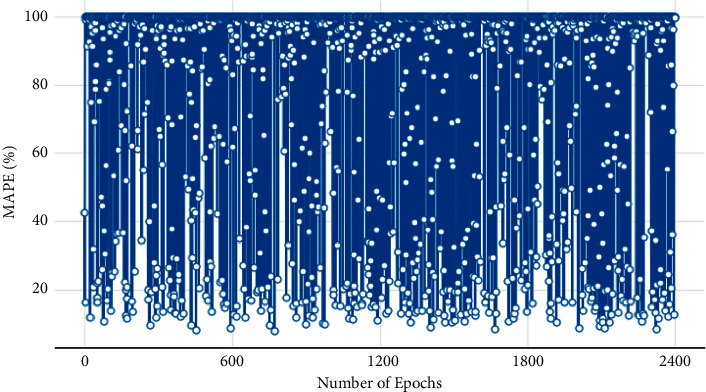
Variation of validation MAPE with number of rounds performed.

**Figure 8:**
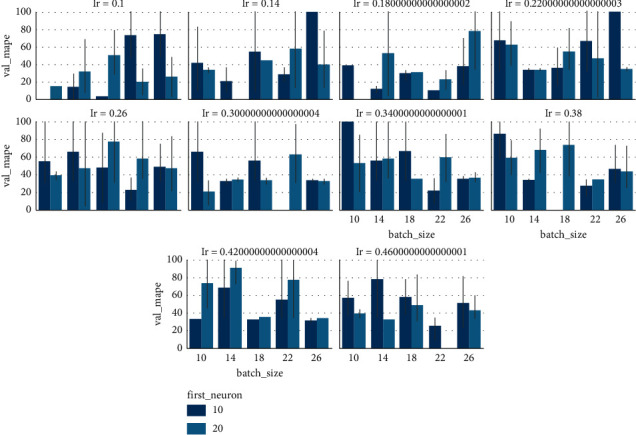
4-dimensional bar plot representing the variation of validation MAPE by using different values for hyperparameters.

The line plot in Figure 7 represents the variation in validation MAPE values with each round during the hyperparameter tuning. In addition, the four-dimensional bar grid plot in Figure 8 is used to visualize how validation MAPE is varying with different parameters. The dark blue bars represent 10 neurons and light blue represents 20 neurons in the first layer. It can be visualized from the bar plot that when “*lr*” is 0.1, the validation MAPE for 10 neurons was significantly lower than 20 neurons. To get the best model, talos. Best_model(“metric: low MAPE”) is used and the best model summary is shown in Table 3. For the test set, the MAPE value for the best model is 8.03. It can be interpreted that the model has good balance between variance and bias.

**Figure 9:**
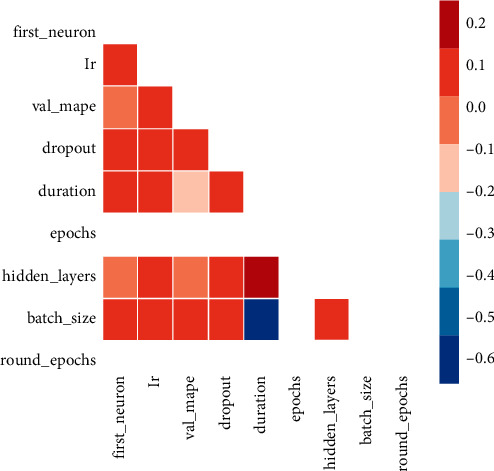
Correlation heat map of hyperparameters.

Moreover, the correlation heat map in Figure 9 represents the correlation between hyperparameters. It can be inferred from the heat map that validation MAPE is highly correlated with duration, number of hidden layers, and “*lr*.”

## 6. Comparative Analysis

**Figure 10:**
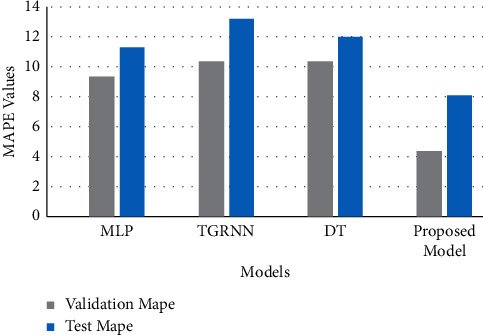
Comparison of MAPE scores of other models with the proposed model.

**Figure 11:**
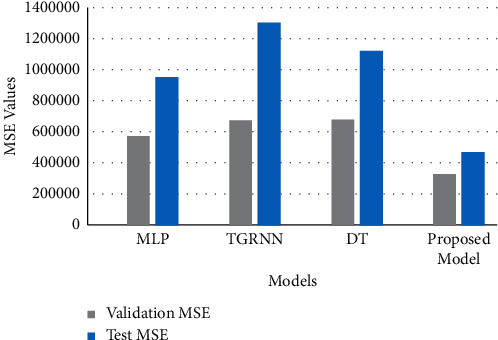
Comparison of MSE scores of other models with the proposed model.

In this section, the proposed model is compared with the already existing models like multilayer perceptron (MLP) with three hidden layers, Decision Tree and Two-stream Gated Recurrent Neural Network. The comparative graph is shown in Figures 10 and 11. It can be clearly interpreted that the proposed model with hyperparameter tuning achieved the lowest MAPE and MSE values for both validation and test sets.

## 7. Conclusions

The work is carried out in three parts: the data extraction and derivation of technical indicators, extensive feature engineering, and stock index price prediction using customized neural network with hyperparameter optimization. We extracted the structured Nifty50 index data and COVID-19 data. From the extracted market data, 9 technical indicators have been derived of which 7 have 4 levels. Five feature selection methods have been applied and averaged coefficient values have been used to select the correct and efficient features. A customized neural network has been built and regressive hyperparameter optimization procedures are applied to achieve faster and optimized predictions. The proposed model has been compared with three other models and results indicate that the proposed model achieved highest accuracy. The MAPE scores for validation and test sets are 4.379007 and 8.03, respectively. In comparison with other models, the proposed model also achieved a low MSE score for both the validation and test sets. Hence, we conclude that the proposed model is a unique approach in comparison to previous works due to the fact that rather than proposing a yet another neural network model, we propose a customized neural network prediction system combined with the extensive feature engineering and hyperparameter optimization procedures to predict stock index prices. Furthermore, the “Daily_Cases” of COVID-19 in India have substantial effect on Nifty50 price changes. Although better results are achieved by the proposed model, this research can further be extended by including the sentiment data from diverse social media platforms, text databases, and news feeds. Furthermore, the model can be extended by analyzing the effect of both fundamental and technical indicators on market.

## Figures and Tables

**Algorithm 1:**
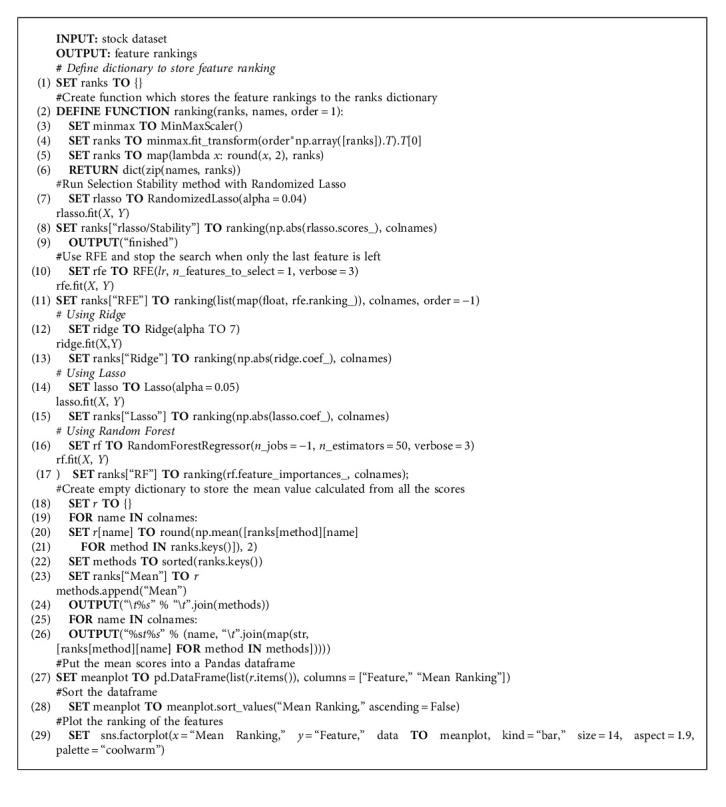
Hybrid feature selection mechanism.

**Algorithm 2:**
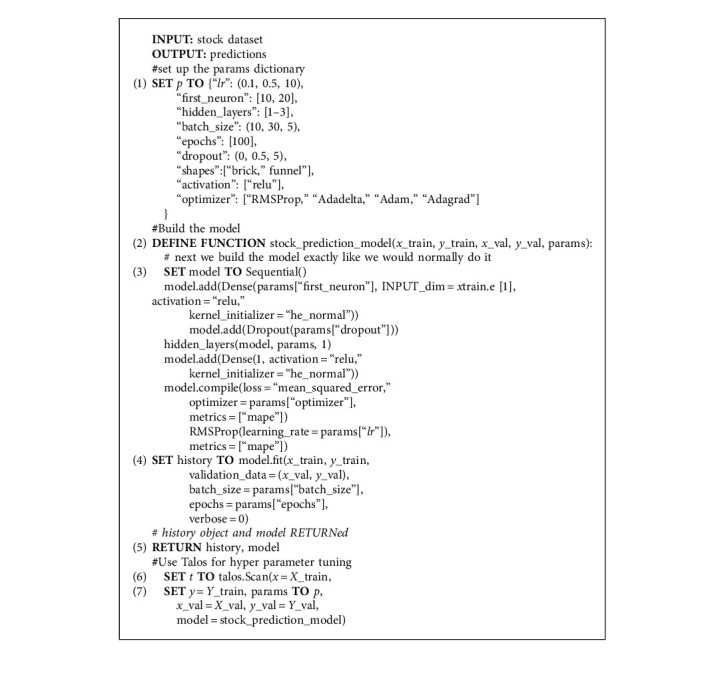
Hyperparameter tuning of ANN.

**Table 1:** Technical indicators, formulas, and levels.

Technical indicators	Formula	Levels
Return	Change=((*C*_*t*_ − *C*_*t*−1_)/*C*_*t*−1_) If change is greater than 0 return=change, else return=0	1
Volatility	SD(change)*∗*sqrt(360)	1
Simple *n* day moving average	*C*_*t*_+*C*_*t*−1_+⋯+*C*_*t*−*n*−1_/*n*	4 (3, 6, 9, 12)
Weighted *n* day moving average (WMA)	(((*n*)*C*_*t*_+(*n* − 1)*C*_*t*−1_+⋯+*C*_*t*−(*n* − 1)_)/(*n*+(*n* − 1)+⋯+1))	4 (3, 6, 9, 12)
Close lag		4 (3, 6, 9, 12)
RSI	100 − (100/(1+(∑_*i*=0_^*n*−1^(UP_*t*−*i*_/*n*))/(∑_*i*=0_^*n*−1^(DW_*t*−*i*_/*n*))))	4 (3, 6, 9, 12)
Moving average convergence/divergence (MACD)	MACD(*n*)_*t*−1_+(2/(*n*+1))*∗*(DIFF_*t*_ − MACD(*n*)_*t*−1_)	4 (3, 6, 9, 12)
Momentum stochastic (*K*%)	((*C*_*t*_ − LL_*t*−(*n* − 1)_)/(HH_*t*−(*n* − 1)_ − LL_*t*−(*n* − 1)_))*∗*100	4 (3, 6, 9, 12)
Commodity channel index (CCI)	((*M*_*t*_ − SM_*t*_)/(0.015*∗D*_*t*_))	4 (3, 6, 9, 12)
Total		30

*Note*. *n* is the time period *n* days ago, *C*_*t*_, *L*_*t*_ , and *H*_*t*_ are the closing, lowest, and highest prices at time *t*, respectively, SD is the standard deviation, SQRT is the square root function, LL_*t*_ and HH_*t*_ are the lowest low and highest high price in last *t* days, respectively, UP_*t*_ and DW_*t*_ are the upward and downward index change at time *t*, DIFF is the EMA(12)_*t*_ − EMA(26)_*t*_, where EMA is exponential moving average, EMA(*p*)_*t*_=EMA(*p*)_*t*−1_+∝(*C*_*t*_ − EMA(*p*)_*t*−1_), where ∝=2/(1+*p*)*p* = 10 in *p*-day EMA. *M*_*t*_=((*C*_*t*_+*L*_*t*_+*H*_*t*_)/3); SM_*t*_=∑_*i*=1_^*n*^(*M*_*t*−*i*+1_/*n*); and *D*_*t*_=∑_*t*=1_^*n*^(|*M*_*t*−*i*+1_ − SM_*t*_|/*n*).

**Table 2:** Hyperparameters with range of values.

Hyperparameters	Values
Learning rate	Range (0.5, 0.1) 10 values
First_neuron	[10, 20]
Hidden layers	[1, 2, 3]
Batch_size	Range (10, 30) 5 values
Epochs	100
Dropout	Range (0, 0.5) 5 values
Shapes	[Brick, funnel]
Activation	ReLU
Optimizer	RMSProp, AdaDelta, Adam, AdaGrad

**Table 3:** Optimized hyperparameters.

Parameters	Values
Start	07/02/21
End	07/02/21
Duration	2 hours and 45 minutes
Round_epochs	100
Loss	618206.8125
MAPE	8.039555
Val_loss	161916.171875
Val_MAPE	4.379007
Activation	ReLU
Optimizer	Adam
Batch_size	10
Dropout	0.0
Epochs	100
First_neuron	10
Hidden layers	2
*Lr*	0.14
Shapes	Funnel
Name	42, dtype: object

## Data Availability

The data used to support the findings of this study are available from the corresponding author upon request.
